# Dual-Modality X-Ray-Induced Radiation Acoustic and Ultrasound Imaging for Real-Time Monitoring of Radiotherapy

**DOI:** 10.34133/2020/9853609

**Published:** 2020-05-26

**Authors:** Wei Zhang, Ibrahim Oraiqat, Hao Lei, Paul L. Carson, Issam EI Naqa, Xueding Wang

**Affiliations:** ^1^Department of Biomedical Engineering, University of Michigan, USA; ^2^Department of Radiation Oncology, University of Michigan, USA; ^3^Department of Mechanical Engineering, University of Michigan, USA; ^4^Department of Radiology, University of Michigan, USA

## Abstract

*Objective*. The goal is to increase the precision of radiation delivery during radiotherapy by tracking the movements of the tumor and other surrounding normal tissues due to respiratory and other body motions. *Introduction*. This work presents the recent advancement of X-ray-induced radiation acoustic imaging (xRAI) technology and the evaluation of its feasibility for real-time monitoring of geometric and morphological misalignments of the X-ray field with respect to the target tissue by combining xRAI with established ultrasound (US) imaging, thereby improving radiotherapy tumor eradication and limiting treatment side effects. *Methods*. An integrated xRAI and B-mode US dual-modality system was established based on a clinic-ready research US platform. The performance of this dual-modality imaging system was evaluated via experiments on phantoms and ex *vivo* and *in vivo* rabbit liver models. *Results*. This system can alternatively switch between the xRAI and the US modes, with spatial resolutions of 1.1 mm and 0.37 mm, respectively. 300 times signal averaging was required for xRAI to reach a satisfactory signal-to-noise ratio, and a frame rate of 1.1 Hz was achieved with a clinical linear accelerator. The US imaging frame rate was 22 Hz, which is sufficient for real-time monitoring of the displacement of the target due to internal body motion. *Conclusion*. Our developed xRAI, in combination with US imaging, allows for mapping of the dose deposition in biological samples *in vivo*, in real-time, during radiotherapy. *Impact Statement*. The US-based image-guided radiotherapy system presented in this work holds great potential for personalized cancer treatment and better outcomes.

## 1. Introduction

As a part of the treatment plan, more than half of all cancer patients receive radiotherapy (RT) which is the main treatment modality for locally advanced cancer [1]. RT is aimed at controlling or killing malignant cells by using high-energy beams of ionizing radiation, which can damage DNA by direct and indirect ionizations of targeted cells [2]. The ionizing radiation is typically delivered by linear accelerators (Linacs) from outside the body to the targeted tissue through external beam radiation therapy (EBRT) [3]. RT can be used alone or in conjunction with other modalities such as surgery and chemotherapy. It is also used in conjunction with immunotherapy to improve its efficacy [4]. It has been demonstrated that RT has the potential to improve the rates of cure of 3.5 million people and provide palliative relief for an additional 3.5 million people [5]. However, this is contingent on achieving the intended treatment effects of eradicating the tumor while sparing surrounding normal tissues. Achieving such desired therapeutic ratio, i.e., the ratio of probabilities of tumor control and unacceptable toxicity, requires that the radiation dose be delivered within less than 5 percent deviation [6, 7]. In clinical settings, however, often the delivered dose to the tumor is limited by exposure of particularly sensitive normal tissues near the treatment region, making a dose control with high precision elusive.

Following the strategy of delivering maximum radiation doses to the targeted area while minimizing the dose to surrounding healthy tissue [8], advanced radiation therapy technologies have been proposed and developed in the past [9, 10]. As an example, intensity-modulated radiotherapy (IMRT) uses sophisticated 3D imaging techniques, such as computed tomography (CT), magnetic resonance imaging (MRI), and positron emission tomography (PET), to obtain the 3D structural information of the tumor and then shape the radiation beams from different orientations accordingly [11]. Although these advanced technologies such as IMRT can solve some of the limitations associated with 3D conformal radiation therapy (3D-CRT) during the planning stage [12, 13], improved conformality and efficiency during delivery remained challenging. More recently, an advanced mode of high-precision RT, volumetric modulated arc RT (VMAT), has been introduced, which uses sophisticated imaging techniques and computer-controlled dynamic multileaf collimation (MLC) through inverse planning optimization to deliver precisely controlled radiation doses to targeted tissue while the radiation beam is continuously reshaped and its dose rate is also optimized to generate highly conformal dose distributions [14, 15]. In practice, large uncertainties still exist in the tumor or target area during time-resolved intrafraction dose delivery and between fractions. These uncertainties, including target positioning and organ mobility as a result of body, cardiac, and respiratory motions, as well as anatomic variations during treatment [16, 17], may significantly alter the doses delivered to both the malignant tumor and adjacent healthy tissue [18, 19]. To eliminate or significantly reduce those uncertainties and improve the treatment accuracy, image-guided radiation therapy (IGRT) has been introduced with an additional time dimension, focusing on utilizing sophisticated imaging technologies to guide the delivery of precise dose in real time during RT [9, 10, 17, 20, 21]. Many modern medical imaging technologies have been investigated to achieve IGRT [22, 23], including nonionizing and noninvasive modalities such as ultrasound (US) [24], video camera, and MRI [25], as well as on-board radiographic imaging such as electronic portal imaging device (EPID) [26, 27], fan beam CT (FBCT), and cone beam CT (FBCT) [28, 29]. However, none of these imaging technologies can achieve, simultaneously and in real time, both the imaging of the morphology and the motion of target tissue and the monitoring of the location of the radiation beam and the local dose deposition.

X-ray-induced radiation acoustic imaging (xRAI) is a novel imaging concept with the potential to map the position and the dose information of the radiation beam in real time during RT, without the need of involving any additional radiation sources [30]. When the high-energy pulsed photon beam generated by Linacs is delivered to the target tissue for therapy, it is absorbed by the tissue under irradiation. The absorbed energy transfers to heat by multielectron collisions, while the rise in temperature in the tissue generates pressure waves as a result of the thermal-acoustic effect [31, 32]. These pressure waves propagating in the tissue can be detected by ultrasonic transducers. These detected X-ray-induced radiation acoustic (XA) signals have their amplitudes proportional to the absorbed X-ray energy. With the XA signals detected by an array of transducers, or via the scanning of a single-element transducer, the spatially distributed X-ray dose deposition in the tissue can be quantitatively mapped [33].

The detection of XA signals produced by clinical Linacs and the imaging of a lead object in chicken breast tissues via tomographic techniques (i.e., XACT) have been achieved first by Xiang et al., which led to a resurgence in this area [34]. In previous publications including those from our group, the fundamental concept of XACT has been described [35], and the ability to form XACT images in pure water in clinically relevant situations and to extract accurate dosimetric information from such images has been demonstrated [30]. More recently, the feasibility of a slow-speed XACT system in mapping the position of the radiation beam and in quantifying the delivered dose has been presented via the experiments on soft-tissue phantoms [36]. Although these exploratory studies are crucial steps toward the development of a clinically applicable xRAI/XACT system, they are all based on in-house-fabricated devices relying on the scanning of a single-element ultrasonic transducer. The mechanical scan of XA signals for tomographic imaging is time-consuming and adds unnecessary blur, especially when the signal-to-noise ratio (SNR) is low and needs to be compensated by extensive signal averaging. In order to enhance the sensitivity in XA signal detection, the transducers used in previous studies all have large element sizes which also lead to limited acceptance angles and hence compromised image quality when the image reconstruction is based on synthetic aperture techniques. Moreover, the xRAI/XACT systems based on in-house-fabricated designs and receiving circuitries are not easily interfaced to commercial US scanners to take the advantage of the state-of-the-art US image processing, management, and display technologies.

To further develop xRAI and promote the translation of the technology to clinical settings of RT, we are exploring the feasibility that the acquisition, processing, and display of xRAI images can be achieved via a commercial US imaging system. Since both xRAI and US involve detection of ultrasonic signals from the target tissue, xRAI may take advantage of the state-of-the-art US imaging technologies. For example, the imaging speed of xRAI can be significantly improved by acquiring data from the parallel US channels each with commercial-grade receiver sensitivity and noise figures. In addition, with the dual-modality arrangement, xRAI and US images of a target can be scanned using the same system, generally along the same viewing angle with essentially the same refraction errors, resulting in naturally coregistered images. The US image can present the morphological tissue structures and motions in the body, as well as functional information such as blood flow and vascular density which are important parameters for treatment planning, while the xRAI image can map and quantify the spatially distributed dose deposition in different biological tissues. Achieving both simultaneously using a combined dual-modality system offers a promising solution to solve the long-standing need for real-time monitoring of beam position and online assessment of delivering dose during RT. To alleviate confusion between tomographic and nontomographic XA approaches, we will use the notion of xRAI instead of XACT for clarity.

In this study, for the first time to the best of our knowledge, xRAI was developed and realized on a clinic-ready research US platform (Vantage, Verasonics) equipped with two low-frequency P4-1 probes (1-4 MHz, Philips). The performance including the spatial resolution of this system for xRAI was first tested by imaging a lead object. Then, the feasibility of the system for IGRT, specially its capability for monitoring the alignment of the X-ray beam with respect to the targeted tissue, was evaluated via experiments on *ex vivo* tissue samples and a rabbit liver model *in vivo*.

## 2. Materials and Methods

### 2.1. Theory of X-Ray-Induced Acoustic Wave

The mathematical model of the X-ray-induced acoustic wave is described using the wave equation [32-34]. The wave equation in the time domain with dose deposition $D\left(r,t\right)$ is given by

(1)∇2−1vs2∂2∂t2pr,t=−βρηthCv∂Dr,t∂t,

where $v_{\text{s}}$ is the speed of sound, β is the volumetric thermal expansion coefficient, $C_{v}$ is the specific heat capacity, and $\eta_{\text{th}}$ is thermal efficiency. Hence, the acoustic pressure detected at the transducer position r and time t can be expressed by

(2)pr,t=14πvs2∫dr′1r−r′Γηthρ∂Dr′,t′∂t′t′=t−r−r′/vs,

where Γ is the Grüneisen parameter defined as

(3)Γ=βKTCvρ,

where $\;K_{T}\;$ is the isothermal bulk modulus.

### 2.2. Experimental Setup and Imaging Method

**Figure 1:**
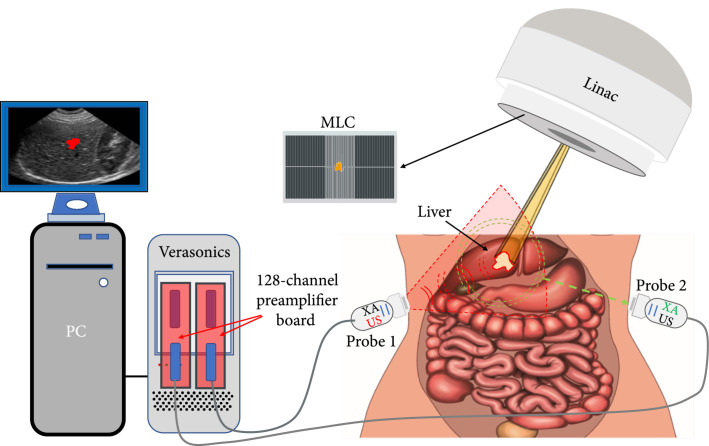
Schematic of an xRAI and US dual-modality imaging system built on a commercially available research US platform. Custom-designed 128-channel preamplifier boards are used to enlarge the XA signals acquired by the probe before sending to the US platform. MLC: multileaf collimation.

The integrated dual-modality imaging system for both xRAI and B-mode US imaging is shown in Figure 1. With 256 parallel channels, the US unit (Vantage, Verasonics) can drive two phase array probes (P4-1, 1-4 MHz, 96 elements, Philips) simultaneously. To generate a compounded image, the two probes acquired signals along different orientations, both facing the center of the sample which was illuminated by the X-ray beam. The center plane of the target and the scanning planes of the probes were adjusted to the same height which had a source-to-axis distance of 100 cm [37]. Water or US couple gel was applied between the probes and the sample for the coupling of ultrasonic waves.

In the xRAI mode, the high-energy X-ray beam from a clinical Linac system (TrueBeam™, Varian) shot from the top. The energy of the photon beam was 6 MV flattening filter free (12.01 Gy/min, 1400 MU/min), the same as the energy used in common clinical RT practice. The pulse duration on this energy level was 4 *μ*s with a 330 Hz pulse repetition rate. The trigger signals from the Linac system, after a delay controlled precisely by a delay generator (DG535, Stanford Research Systems), were sent to the Verasonics US system for synchronization. With the XA signals detected by the system, xRAI images were reconstructed and displayed in real time. In the US mode, the probes driven by the same system acquired B-mode images from the same sample, also in real time. The US imaging mode ran for every 15 triggers, and the xRAI mode ran for every trigger.

One of the biggest challenges in realizing xRAI by using a commercial US system is the extremely low XA signal amplitude, which was at the nV level when working with the clinical level photon energy, and the limited sensitivity and dynamic range of the commercial US system. In our system, when working in the xRAI mode, the XA signals detected by the P4-1 probes were first amplified by a custom-built multichannel preamplifier board with 40 dB gain before being transferred to the Verasonics US unit. For the setting of the Verasonics unit, time gain compensation (TGC) with up to 40 dB gain was applied to enhance the signal amplitudes and compensate the attenuation increasing with the depth. The DC and the low frequency background drift of the detected signals were removed by a high-pass filter with 80 kHz cutoff frequency. A programmable-gain amplifier (PGA) and a low-noise amplifier (LNA) were also used to further amplify the signals so that the XA signal amplitudes could be in the detectable range of the digitizer in the Verasonics unit. Two high-pass filters right after the PGA and LNA were disabled to achieve a flat frequency response all the way down to DC. After this signal processing, the XA signals were digitized by a 14-bit A/D with a 10 MHz sampling rate. To further enhance the SNR, 300 times signal averaging was applied before image reconstruction. Therefore, each frame of the 2D xRAI image was generated in 300/330=0.91 seconds. With the XA signals acquired by each of the two P4-1 probes, an xRAI image was reconstructed using the basic delay-and-sum method. Then, the two images from the two probes were compounded after image coregistration by considering the positions and the orientations of the two probes.

In the US imaging mode, two sets of B-mode US images were produced by using the two probes, following the same signal processing procedure with only 20 dB TGC gain and no signal averaging. Then, the two sets of US images were compounded after image coregistration between the two probes. The sampling rate of the digitizer was set to 10 MHz. With the US imaging running once for every 15 triggers, the imaging frame rate was 330/15=22 Hz. Since the two imaging modalities in our system share the same probes, the xRAI and the US images are naturally coregistered and can be easily fused together as a combined image.

### 2.3. Calibrating the Imaging Resolution

A lead block was used as the imaging target to examine the spatial resolution of the xRAI system. The 20 mm×20 mm×20 mm size lead cube was put 40 mm away from the surface of the probe. Both the probe and the lead block were immersed in water for acoustic coupling. The X-ray field with a size of 20 mm×20 mm covering the entire cube shot from the top.

### 2.4. Demonstrating the Dual-Modality Imaging Capability

To demonstrate the feasibility of our system for xRAI and US dual-modality imaging, a porcine gel phantom (a cylinder with diameter of 100 mm and height of 30 mm) containing three lard blocks (10 mm in length, 10 mm in width, and 20 mm in height) was imaged. The X-ray field with a size of 10 mm×10 mm targeted at each of the lard blocks. Image coregistration was later applied to fuse the images from the two modalities together to generate a combined image showing both the structure and the X-ray dose deposition in the phantom.

### 2.5. Tracking the Size of X-Ray Beam

To verify the performance of the system in measuring the size of the X-ray beam, Colza oil was filled in a water tank with 40 mm in depth. In this study, only one US probe was employed for imaging. The US probe was immersed in oil for acoustic coupling. The X-ray beam with different sizes illuminated on the phantom vertically from above. The size of the X-ray beam along the lateral direction of the US probe was fixed at 10 mm, while the size along the axial direction of the US probe changed from 10 mm to 20 mm, with a constant step of 1 mm, shaped by controlling the MLC. The near boundary of the X-ray beam was fixed at 40 mm distance from the probe. For the X-ray beam with each size, 5 independent xRAI images were acquired for further statistical analysis.

### 2.6. Monitoring of Sample Motion with respect to X-Ray Beam *Ex Vivo*

An experiment on *ex vivo* fresh rabbit livers was conducted to examine the feasibility of the dual-modality imaging system in monitoring the misalignment between the X-ray beam and the treatment target. The two probes were oriented at 0° and 90°, respectively, both facing the center of the sample which was illuminated by the X-ray beam. The X-ray beam delivered to the sample was a 15 mm×15 mm square. The sample, driven by a translation stage with a stepper motor, was continuously moved along the direction marked by the blue arrow, with an average motion speed of 2.5 mm/s (similar to the situation of the motion of the human liver due to the breath). Over a time period of 11 seconds, the sample moved a total distance of 27.5 mm (also similar to the scale of the motion of the human liver due to the breath). The relative position of the X-ray dose deposition with respect to the probe was fixed. The US mode and the xRAI mode were performed separately during the motion of the sample.

### 2.7. Real-Time Tracking of the Movement of Organ with respect to the Dose Deposition *In Vivo*

An *in vivo* experiment performed using a rabbit model was conducted to examine the feasibility of the dual-modality imaging system in real-time tracking of the movement of the targeted tissue with respect to the position of the treatment X-ray beam. All the animal handling procedures were approved by the Institutional Animal Care and Use Committee at the University of Michigan (Protocol PRO00008698, “Investigation of radiation acoustics and optics for real-time guidance in radiotherapy”). Three New Zealand white rabbits (3.5-4 kg) were involved in this study. The animals were anesthetized with a mixture of ketamine (40 mg/kg) and acepromazine (0.5 mg/kg) via intramuscular (IM) injection. Following this, anesthesia was maintained with 1.5% isoflurane and oxygen using a V-Gel® (J1350D, Jorgensen Laboratories, Loveland, CO). The anesthesia level was evaluated through continuous monitoring of the heart rate and respiratory rate. An adjustable water-circulating heating pad (TP-700, Stryker Corporation, Kalamazoo, MI) was used to keep the body temperature stable. The maximum delivered X-ray dose to each rabbit did not exceed 20 Gy.

**Figure 2:**
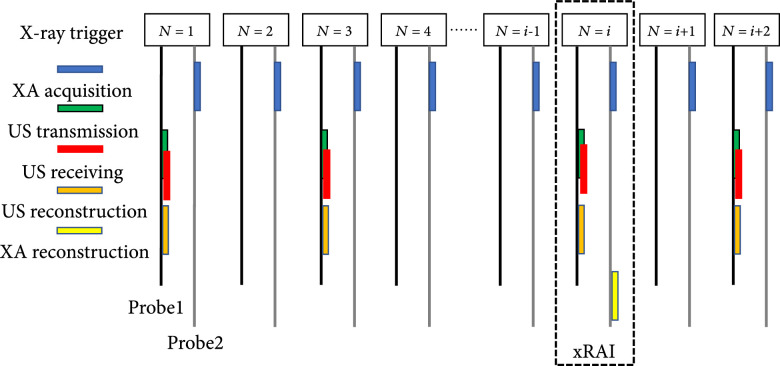
Timing sequence for xRAI and US real-time dual-modality imaging of an in vivo rabbit liver. The xRAI image was reconstructed with i times average.

The rabbit was fixed with a custom-built rabbit holder during image acquisition. The high-energy X-ray beam with 20 mm×20 mm beam size from a research Linac platform (Linatron, Varex Imaging) shot the rabbit liver horizontally. The energy of the photon beam was 9 MeV with 4 *μ*s pulse duration and 44 Hz repetition rate (1.36 Gy/min). Two US probes (P4-1, 1-4 MHz, 96 elements, Philips) were fixed together and placed on the right side of the body close to the liver. Following the timing sequence shown in Figure 2, two probes were working in the US mode and the xRAI mode, respectively. The relative position of the probe with respect to the beam was fixed. The trigger signals from the Linatron, after a delay controlled precisely by a delay generator (DG535, Stanford Research Systems), were sent to the Verasonics US system for synchronization. In the xRAI mode, 440 times signal averaging was applied before image reconstruction. Therefore, each xRAI image acquired *in vivo* took 10 seconds. The US image was acquired for every 2 triggers, at a frame rate of 22 Hz. Following the method described above, xRAI and B-mode US images of the liver were acquired at the same time, reconstructed both in real time, and displayed simultaneously on the computer screen. The position of the beam was aligned by a laser calibration system. The X-ray dose was calibrated with an ionization chamber (Exradin A16 Ion Chamber, Standard Imaging) before each animal experiment.

## 3. Results

### 3.1. Calibrating the Imaging Resolution

**Figure 3:**
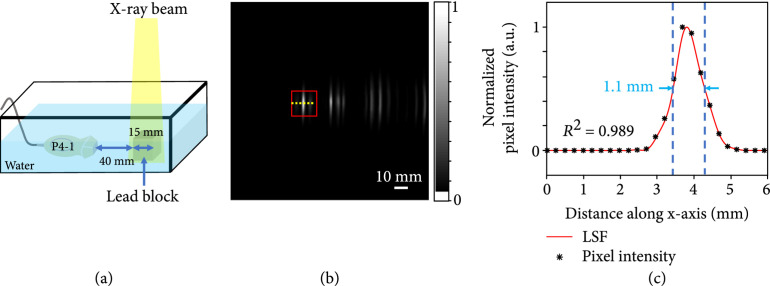
Calibration of the axial spatial resolution of the xRAI system. (a) Experimental setup for quantifying the axial spatial resolution of the xRAI system by imaging a lead block. (b) xRAI image of the lead block. The red square marks the front side of the block. (c) Normalized pixel intensities along the yellow dashed line in xRAI image in (b). The fitting curve shows the line spread function with a FWHM of 1.1 mm.

Using the setup shown in Figure 3(a), the acquired xRAI image of a lead block is shown in Figure 3(b). The front side of the block shown by the first vertical line from the left is enclosed by a red square. The back side of the block is shown by the next vertical line, apparently further than 20 mm to the right of the first vertical line because of the low, 1200 m/s speed of sound in lead compared with the 1540 m/s assumed by the imaging system. The normalized intensity profile along the dotted line in the red box is presented in Figure 3(c), where the dots show the pixel intensities along the dotted line. The curve shows the fitted line spread function (LSF) ($R^{2}=0.989$), which has a quantified full width at half maximum (FWHM) of 1.1 mm, suggesting that the axial spatial resolution of the current xRAI system is better than 1.1 mm by virtue of the deconvolution process.

### 3.2. Demonstrating the Dual-Modality Imaging Capability

**Figure 4:**
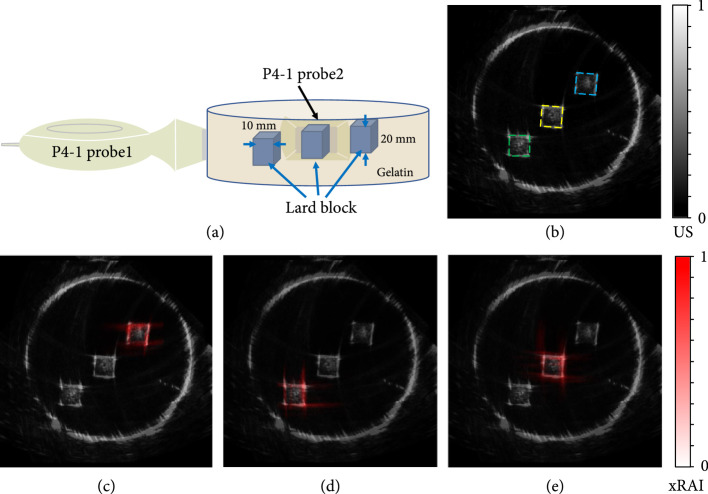
Demonstration of the feasibility for xRAI and US dual-modality imaging via imaging of lards in gel: (a) experimental setup; (b) grayscale US image showing the structure of the phantom with three lard blocks to be targeted separately by the X-ray beam; (c-e) pseudocolor xRAI images, superimposed on the grayscale B-mode image, showing in red the boundaries of the separately deposited X-ray dose in the top through bottom lard blocks, respectively.

The experimental setup for dual-modality imaging of a porcine gel phantom containing three lard blocks is shown in Figure 4(a). The grayscale B-mode US image shown in Figure 4(b) presents the structure of the phantom in the imaged plane, including the boundary of the porcine gel and the locations of the lard blocks. Figures 4(c)-4(e) show the pseudocolor xRAI images of the same phantom when the X-ray beam is targeted at each of the lard blocks, respectively, where each xRAI image is superimposed on the grayscale B-mode US image. Since the xRAI images and the B-mode US image are coregistered naturally, they can be easily combined. In each xRAI image, the boundaries of the X-ray dose deposition are clearly mapped. With the phantom structure shown by the US image, each combined image presents the exact location and distribution of the X-ray dose deposition with respect to the position of each of the targeted lard blocks.

### 3.3. Tracking the Size of X-Ray Beam

**Figure 5:**
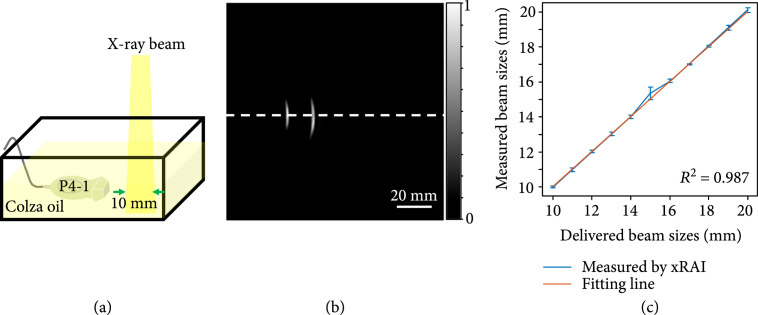
xRAI result showing the capability of measuring beam size: (a) experimental setup for measuring the X-ray beam size; (b) xRAI image of an X-ray beam with a size of 10 mm×17 mm; (c) Comparison of quantified X-ray beam sizes measured in xRAI images and the actual beam sizes controlled by MLC. The red solid line shows a linear fitting ($R^{2}=0.987$).

Using the experimental setup shown in Figure 5(a), xRAI images were obtained when X-ray beams with different sizes were delivered to a tank filled with colza oil. The xRAI image in Figure 5(b) was acquired when the X-ray beam size was 10 mm×17 mm. The image intensity profile along the white dashed line was extracted to quantify the beam size. With the results from 11 beams with different sizes ranging from 10 mm to 20 mm, the beam sizes quantified from xRAI images are compared with the delivered beam sizes, as shown in Figure 5(c). For each delivered beam size, the mean and the standard deviation of xRAI measurements are shown. A linear fitting was performed, and $R^{2}=0.987$ was achieved, demonstrating that xRAI, by mapping the deposited X-ray dose in the sample, can measure the beam size with a good accuracy. The change in beam size that equals to the minimal size variable of a clinical linear accelerator (1 mm) can be detected, with a maximum deviation of 0.34 mm and a mean standard deviation of 0.09 mm.

### 3.4. Monitoring of Sample Motion with respect to X-Ray Beam

**Figure 6:**
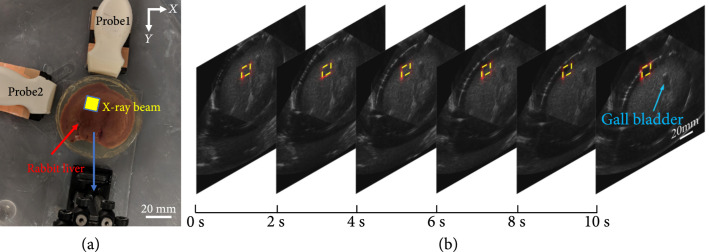
xRAI and US dual-modality imaging of an *ex vivo* rabbit liver, demonstrating the capability of tracking tissue movement with respect to the X-ray beam. (a) The setup for validating the capability of the system in monitoring of misalignment between the X-ray beam and the treatment target (blue arrow indicates the direction of sample motion). (b) The xRAI and US combined images at different time points (0, 2, 4, 6, 8, and 10 seconds) during continuous imaging over a time period of 11 seconds. In each combined image, the xRAI image in pseudocolor presenting the location of the X-ray dose deposition (marked by the yellow dashed box) is superimposed on the US image in grayscale showing the tissue structure.

Figure 6 shows the xRAI and US dual-modality imaging result from a rabbit liver *ex vivo*. Two images acquired independently by the two probes shown in Figure 6(a) were fused. The pseudocolor xRAI and grayscale US combined images were acquired at different time points during a period of 0-11 seconds. In this time period, the sample was moved continuously while the position of the X-ray beam was kept static. The motion of the sample with respect to the X-ray beam was imaged successfully, as shown in Figure 6(b). The position of the delivered X-ray beam is marked by the yellow dashed box. The frame rate of US imaging at 22 Hz is sufficient for tracking the continuous tissue motion. Although the frame rate of our current xRAI is relatively slow (1.1 frames per second) due to the need for extensive signal averaging, it is acceptable for confirming the position of a static X-ray beam during continuous imaging. In other words, even though the xRAI frame rate is relatively low, the relative position between the delivered X-ray beam and the treatment target can be correctly displayed, benefiting from the 22 Hz US imaging speed. The video of this dual-modality imaging result from an *ex vivo* rabbit liver model is shown in Movie S1 in Supplementary Materials.

### 3.5. *In Vivo* Tracking of the Movement of Organ with respect to the Dose Deposition

**Figure 7:**
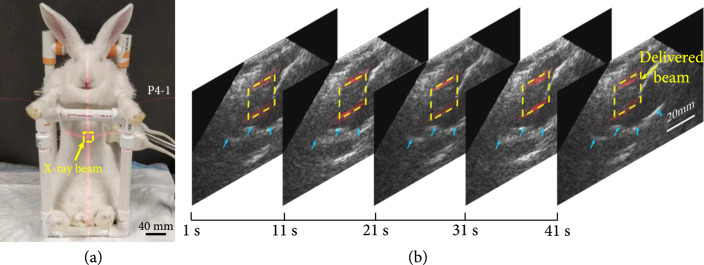
xRAI and US real-time dual-modality imaging of an *in vivo* rabbit liver, demonstrating the capability for tracking tissue movement with respect to the X-ray beam. (a) The setup for validating the capability of the system in real-time tracking the movement of the target tissue with respect to the position of the X-ray beam illuminating on a live rabbit. (b) The xRAI and US combined images at different time points (1, 11, 21, 31, and 41 seconds) during the real-time imaging over a period of 50 seconds. In each combined image, the xRAI image in pseudocolor presenting the location of the X-ray dose deposition (marked by the yellow dashed box) is superimposed on the US image in grayscale showing the tissue structure.

As shown in Figure 7(a), the rabbit was fixed with a custom-built rabbit holder during image acquisition. The position of the beam was aligned by a laser calibration system shown with red color. The result of xRAI and US dual-modality imaging on a rabbit model *in vivo* is shown in Figure 7(b). The pseudocolor xRAI images mapping the area of X-ray dose deposition were superimposed on the US images presenting the tissue structure including the target liver. As indicated by the blue arrows, the edge of the rabbit liver can be distinguished clearly in the US images. With a frame rate of 22 Hz, the gray scale US images can track the motion of the liver caused by the heartbeat and the breath in real time and, when combined with the xRAI images, can track the tissue movement with respect to the X-ray beam. The video of this real-time dual-modality imaging result from the *in vivo* rabbit liver model is shown in Movie S2 in Supplementary Materials.

## 4. Discussion

The goal of this research is to increase the precision of radiation delivery during RT by tracking the movements of the tumor and other tissues due to respiratory and other body motions and the position of the X-ray beam relative to those tissues while the treatment is being delivered in real time. To achieve this goal and solve the long-standing challenge in RT, a new xRAI and US dual-modality imaging system was developed on a clinic-ready research US platform. Its feasibility in quantitative mapping of the X-ray dose in biological samples and its capability for tracking the geometrical and morphological misalignments of the X-ray beam with respect to the target tissue in real time were investigated. As demonstrated by the experimental results from the phantoms and *ex vivo* and *in vivo* rabbit liver models, the anatomical information of the tissue can be obtained through US imaging, while the xRAI image acquired at the same time can provide the dosimetric information of the X-ray dose deposition in local tissue. This additional information is highly valuable during external beam RT since it can greatly improve the accuracy in tumor targeting and largely mitigate the undesirable collateral damage to surrounding normal tissues, thereby leading to a better patient outcome. Taking advantages of the large number of parallel channels and the excellent controllability of the research US platform, xRAI and US imaging of the rabbit liver can be achieved simultaneously. The 22 Hz frame rate of the US imaging is sufficient to track the tissue motion due to heartbeat and breath. Although the current imaging speed of xRAI (1.1 Hz), limited by the SNR in detecting the XA signals, is much lower than US imaging (22 Hz), it works well for confirming the position of a static X-ray beam during continuous imaging. By combining the xRAI and US images, which are naturally coregistered, monitoring of the misalignment of the X-ray field with respect to the target tissue can be achieved continuously, in a near real-time fashion.

With the unique capability in mapping the radiation and quantifying the dose deposition in biological samples *in vivo* in real time, the xRAI technology presented in this work renders a promising method for IGRT. Building xRAI and US dual-modality imaging by utilizing a commercial US unit can largely accelerate the technology development and speed up the translation of xRAI to clinic. Using the same US probe and the large number of parallel channels of the US unit for detection of XA signals not only improves the imaging speed but also facilitates a natural coregistration between the xRAI image mapping the X-ray dose and the US image rendering the tissue structure. Despite many advantages presented by the dual-modality design, xRAI, however, suffers from the limited detection sensitivity of the commercial US unit. As discussed in Supplementary Materials, both the amplitude (~10 nV) of the XA signals from soft tissues acquired by the P4-1 probe and the SNR (10 : 1) are very low. To enhance the signal level and fully utilize the dynamic range of the 14-bit digitizer in the Verasonics US unit, a custom-designed 128-channel preamplifier board was used to enlarge the amplitudes of both XA signals and noise from the P4-1 probe before sending them to the US unit. To achieve sufficient SNR when imaging soft-tissue samples *ex vivo* and *in vivo*, extensive signal averaging is performed which inevitably reduced the imaging speed. As shown in this study, when working with a clinical Linac, the frame rate of the dual-modality imaging is over 22 Hz for B-mode US imaging but only 1.1 Hz for xRAI which is limited by the extensive signal averaging over 300 times. Suffering from the ten times lower repetition rate of the research Linatron platform, the *in vivo* study performed on a rabbit model only achieved 0.1 frame per second for xRAI. Since the pulse duration of the X-ray source is about 4 *μ*s, the produced XA signals are more dominated at the very low frequency range around 250 kHz. To better detect these low frequency signals, we used P4-1 probes which are one of the lowest frequency probes that are commercially available. However, the P4-1 probe still cannot cover the very low frequency range of the XA signals. In the future, a custom-designed US unit with better sensitivity at the lower frequency range that can fully cover the 250 kHz frequency should provide improved SNR in detecting XA signals and improved frame rate in xRAI.

Another limitation of the xRAI realized by the current system is its relatively low spatial resolution. As demonstrated by the quantified imaging results from the lead block, the axial resolution in the xRAI mode is 1.1 mm, which is also much lower than the axial resolution of 0.37 mm in the US mode determined by the center frequency of the probe. The low spatial resolution is also caused by the low frequency of the XA signals produced by the X-ray pulses with a long pulse duration of 4 *μ*s. One solution to improve the spatial resolution of xRAI is to perform a deconvolution to remove the effect due to the long X-ray pulse, as performed in this study. This method, however, cannot completely solve the problem when the probe used for xRAI has limited bandwidth. In the future, when the X-ray pulse duration from the Linac system could be further reduced, the XA signal in the tissue can be generated with not only higher efficiency but also higher frequency and broader bandwidth. Then, a probe working at higher frequency with broader bandwidth can be employed to largely improve the spatial resolution of xRAI.

Despite the fact that both the imaging speed and the image quality of xRAI still need to be improved, the current results from our developed dual-modality prototype imaging system and the proof of concept experiments on the animal models are highly encouraging and, for the first time, demonstrate the feasibility of xRAI clinical implementation in RT by real-time monitoring the misalignment between the targeted tumor and the delivered beam. The xRAI presented in this work can potentially provide the real-time treatment guidance for RT and holds a great potential to optimize the treatment outcome without interrupting the treatment procedure.

## Data Availability

All data are available within the Article and Supplementary Files, or available from the authors upon request.

## Supplementary Materials

Supplementary materials Figure S1: XA signals detected by a transducer element in the P4-1 probe driven by the Verasonics US unit. (A) The noise level of a transducer element of the P4-1 probe after 94 dB gain provided by the US unit. (B) An A-scan XA signal embedded in noise acquired from a soft-tissue sample by a P4-1 transducer element, where both the signal and the noise were amplified by a total gain of 134 dB (i.e., 94 dB from the US unit and 40 dB from the preamplifier board). No signal averaging is performed. (C) An A-scan XA signal acquired from a soft-tissue sample by a P4-1 transducer element which was extensively averaged over 440 X-ray pulses after a total amplification of 134 dB. An SNR of 2 : 1 was achieved by signal averaging. Movie S1: xRAI and US dual-modality imaging of an ex vivo rabbit liver, demonstrating the feasibility of the imaging system in tracking the tissue movement with respect to the X-ray beam. Movie S2: xRAI and US real-time dual-modality imaging of a rabbit liver in vivo, demonstrating the feasibility of the imaging system in tracking the tissue movement with respect to the X-ray beam.
