# Supplementary material for: Dual-Modality X-Ray-Induced Radiation Acoustic and Ultrasound Imaging for Real-Time Monitoring of Radiotherapy
Source: BME Front. 2020 May 26;2020:9853609. doi: 10.34133/2020/9853609 (PMC10521688; doi:10.34133/2020/9853609)
Supplement: Supplementary materials — Figure S1: XA signals detected by a transducer element in the P4-1 probe driven by the Verasonics US unit. (A) The noise level of a transducer element of the P4-1 probe after 94 dB gain provided by the US unit. (B) An A-scan XA signal embedded in noise acquired from a soft-tissue sample by a P4-1 transducer element, where both the signal and the noise were amplified by a total gain of 134 dB (i.e., 94 dB from the US unit and 40 dB from the preamplifier board). No signal averaging is performed. (C) An A-scan XA signal acquired from a soft-tissue sample by a P4-1 transducer element which was extensively averaged over 440 X-ray pulses after a total amplification of 134 dB. An SNR of 2 : 1 was achieved by signal averaging. Movie S1: xRAI and US dual-modality imaging of an ex vivo rabbit liver, demonstrating the feasibility of the imaging system in tracking the tissue movement with respect to the X-ray beam. Movie S2: xRAI and US real-time dual-modality imaging of a rabbit liver in vivo, demonstrating the feasibility of the imaging system in tracking the tissue movement with respect to the X-ray beam. [file 9853609.f1.zip › supplementary material.docx]

Title

Dual-modality x-ray induced radiation acoustic and ultrasound imaging for real-time monitoring of radiotherapy

Radiation acoustic for radiotherapy monitoring

**Authors**

Wei Zhang,^1†^ Ibrahim Oraiqat,^2†^ Hao Lei,^3^ Paul L. Carson,^4^ Issam EI Naqa,^2^* Xueding Wang^1,4^*

**Affiliations**

^1^Department of Biomedical Engineering, University of Michigan, United States.

^2^Department of Radiation Oncology, University of Michigan, United States.

^3^Department of Mechanical Engineering, University of Michigan, United States.

^4^Department of Radiology, University of Michigan, United States.

^*^Corresponding authors: [ielnaqa@med.umich.edu](mailto:ielnaqa@med.umich.edu) and [xdwang@umich.edu](mailto:xdwang@umich.edu).

^†^These authors contributed equally to this work.

**Signal processing and image reconstruction**

The acoustic signals detected by P4-1 probe were first amplified by a custom-built multi-channels preamplifier board with 40 dB gain before being transferred to the Verasonics US unit. In the Verasonics unit, a time gain compensation (TGC) amplifier with up 40 dB gain was applied to enhance the signal amplitudes and compensate the attenuation increasing with the depth. A programmable-gain amplifier (PGA) with 30 dB and a low noise amplifier (LNA) with 24 dB were also used to further amplify the signals. A total gain of 134 dB was applied to enhance to signal so that the XA signal amplitudes could be in the detectable range of the digitizer.

Due to the limited bandwidth of the probe, only the signal from the boundary of the beam can be detected by the dual-modality imaging system. The digitized acoustic signal was first deconvoluted with the impulse response induced by the x-ray beam, which can help to eliminate the decrease of spatial resolution caused by the 4-µs x-ray pulse duration. A conventional delay-and-sum reconstruction algorithm that integrated in the Verasonics system was applied to reconstruct the xRAI images in real time.

**Quantification of the detection sensitivity**

In the Verasonics Vantage ultrasound (US) unit, an integrated 14-bit digitizer is used to sample the signal with a dynamic range from -1 V to 1 V. The estimated XA wave pressure at the front of the P4-1 probe is 10 mPa which induces a voltage in each transducer element of about 10 nV. The estimated noise level in each transducer element of the P4-1 probe is 100 nV (i.e. SNR=1:10). After the 94 dB maximum gain provided by the Verasonics US unit, the amplitudes of signal and noise sent to the digitizer are only about 0.5 mV and 5 mV, respectively, which do not fully utilize the ±1 V dynamic range of the digitizer. **Fig.S1A** shows the noise level of a transducer element of the P4-1 probe driven by the Verasonics US unit with full 94 dB gain.

In this study, to enhance the sensitivity in xRAI, a custom designed multi-channel preamplifier board was employed to amplify the XA signals received by the P4-1 array before sending to the US unit. After amplification by the 40 dB preamplifier board, the amplitude levels of the XA signal and the noise sent to the digitizer are about 50 mV and 500 mV, respectively. The ±500 mV noise amplitude level well utilizes the dynamic range of the digitizer (about 50%), leading to good sensitivity for signal detection. **Fig.S1B** shows an example A-scan XA signal embedded in noise acquired from a soft-tissue sample. This A-scan signal was acquired by a transducer element in the P4-1 array, and then digitized in the US unit after a total gain of 134 dB (i.e. 94 dB offered by the US unit and an additional 40 dB provided by the preamplifier board).

To achieve sufficient SNR when imaging soft-tissue samples, the amplified signals embedded in noise need to be averaged extensively over a large number of x-ray pulses. For example, in the *in vivo* experiments on the rabbit liver model, a signal average over 440 x-ray pulses was performed, which improved the SNR from 1:10 to 2:1 ($\sqrt{440}$=21 times). **Fig.S1C** shows an example A-scan XA signal acquired from a soft-tissue sample by a P4-1 transducer element after 440 signal averaging. The SNR of 2:1 for channel data is sufficient for xRAI imaging, considering that the contrast-to-noise ratio can be further enhanced by the delay-and-sum based image reconstruction where a dynamic focus is achieved with the XA signals acquired at multiple channels.


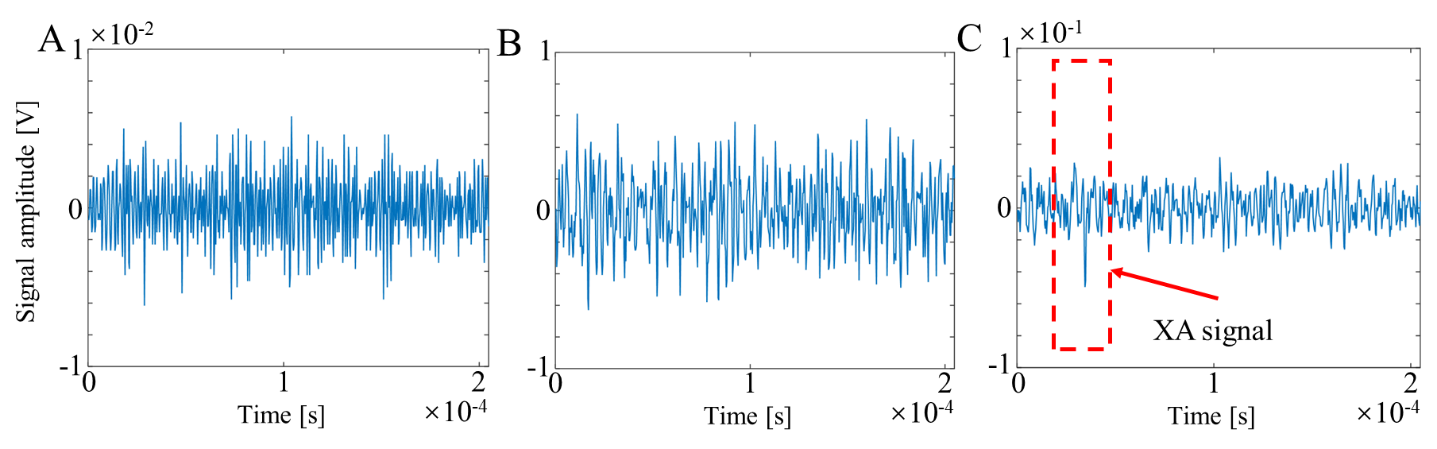


***Figure.S1.*** *XA signals detected by a transducer element in the P4-1 probe driven by the Verasonics US unit. (****A****) The noise level of a transducer element of the P4-1 probe after 94 dB gain provided by the US unit. (****B****) An A-scan XA signal embedded in noise acquired from a soft-tissue sample by a P4-1 transducer element, where both the signal and the noise were amplified by a total gain of 134 dB (i.e. 94 dB from the US unit and 40 dB from the preamplifier board). No signal averaging is performed. (****C****) An A-scan XA signal acquired from a soft-tissue sample by a P4-1 transducer element which was extensively averaged over 440 x-ray pulses after a total amplification of 134 dB. An SNR of 2:1 was achieved by signal averaging.*

***Movie S1.*** *xRAI and US dual-modality imaging of an ex vivo rabbit liver, demonstrating the feasibility of the imaging system in tracking the tissue movement in respect to the x-ray beam.*

***Movie S2.*** *xRAI and US real-time dual-modality imaging of a rabbit liver in vivo, demonstrating the feasibility of the imaging system in tracking the tissue movement in respect to the x-ray beam.*
